# PDIA6, which is regulated by TRPM2-AS/miR-424-5p axis, promotes endometrial cancer progression via TGF-beta pathway

**DOI:** 10.1038/s41419-023-06297-8

**Published:** 2023-12-14

**Authors:** Pengling Wang, Tianli Zhang, Nan Jiang, Kun Wang, Liping Feng, Ting Liu, Xingsheng Yang

**Affiliations:** 1 Department of Obstetrics and Gynecology, Qilu Hospital of Shandong University, Jinan, Shandong 250012 People’s Republic of China; 2 Department of Gynecology, The Second Hospital of Shandong University, Jinan, Shandong 250033 People’s Republic of China

**Keywords:** Endometrial cancer, Endometrial cancer

## Abstract

PDIA6 have been reported to be involved in a variety of cancers, however, the underlying role in endometrial cancer is still unclear. In this study, we aimed to study the function of PDIA6 in endometrial cancer. Firstly, we verified that PDIA6 was significantly upregulated in endometrial cancer, which was correlated with the progression of endometrial cancer patients. Furthermore, we identified PDIA6 significantly altered the ability of endometrial cancer cells to proliferate and metastasize. In addition, our result illustrated the oncogene effects of PDIA6 in promoting malignant biological behavior of endometrial cancer cells by regulating TGF-β pathway and being modulated by TRPM2-AS/miR-424-5p axis for the first time. Taken together, this study suggested that PDIA6 may be a new candidate target for endometrial cancer therapy.

## Introduction

Endometrial cancer (EC) is one of the most common gynecological malignancies [1]. The pathogenesis of endometrial cancer is complicated. Lynch Syndrome and Cowden Syndrome genetic predisposition, polycystic ovary syndrome, tamoxifen use, infertility, diabetes and obesity have been identified as clear risk factors [2–4]. EC patients diagnosed with an early stage usually have a good prognosis [5]. However, for endometrial cancer patients diagnosed as clinically aggressive, the prognosis is poor. Although the chemotherapy, radiotherapy or surgical techniques of endometrial cancer have made great progress in recent years, the survival rate of patients with advanced endometrial cancer is still unsatisfactory. Therefore, there is an urgently need to develop more effective endometrial cancer diagnosis and treatment strategies.

Protein Disulfide Isomerase Family A Member 6 (PDIA6) is a member of the protein disulfide isomerase (PDI) family. Large number of studies have shown that PDIA6 is related to the development of a variety of cancers. For example, PDIA6 could modulate proliferation, invasion, apoptosis and autophagy via the MAP4K1/JNK signaling pathway or the Wnt/β-catenin signaling pathway [6, 7]. Moreover, higher PDIA6 could promote pancreatic cancer progression and immune escape through CSN5-mediated deubiquitination of β-catenin and PD-L1 [8]. However, the effects of PDIA6 in endometrial cancer has not yet been fully established.

In this study, we indicated that PDIA6 was significantly overexpressed in endometrial cancer tissues and correlated with the ability of endometrial cancer cells to proliferation and metastatic properties, so it was selected for further study. Then we aimed to investigate the regulatory mechanism of PDIA6 in endometrial cancer cells. We hypothesized that PDIA6 could promote the growth and metastasis of endometrial cancer cells by regulating TGF-β pathway and being regulated by TRPM2-AS/miR-424-5p axis. Our results may provide a better molecular therapeutic target for the treatment of endometrial cancer.

## Materials and methods

### Tissue specimens

A total of 53 human EC tissues and 10 normal tissues samples were obtained from the Department of Pathology, Qilu Hospital of Shandong University, from January 2013 to December 2016. In addition, a total of 32 pairs of fresh EC tissues and paraneoplastic non-tumor tissues were obtained from the Department of Pathology, Qilu Hospital of Shandong University, from October 2019 to December 2020. All patients were pathologically diagnosed as endometrial cancer by three pathologists. All the selected patients had complete clinical and pathological data and follow-up information. All patients signed the informed consent for the use of samples, and the study was approved by the Ethics Committee of the Qilu Hospital of Shandong University.

## Results

### PDIA6 expression is upregulated in endometrial cancer and correlated to progression

**Fig. 1:**
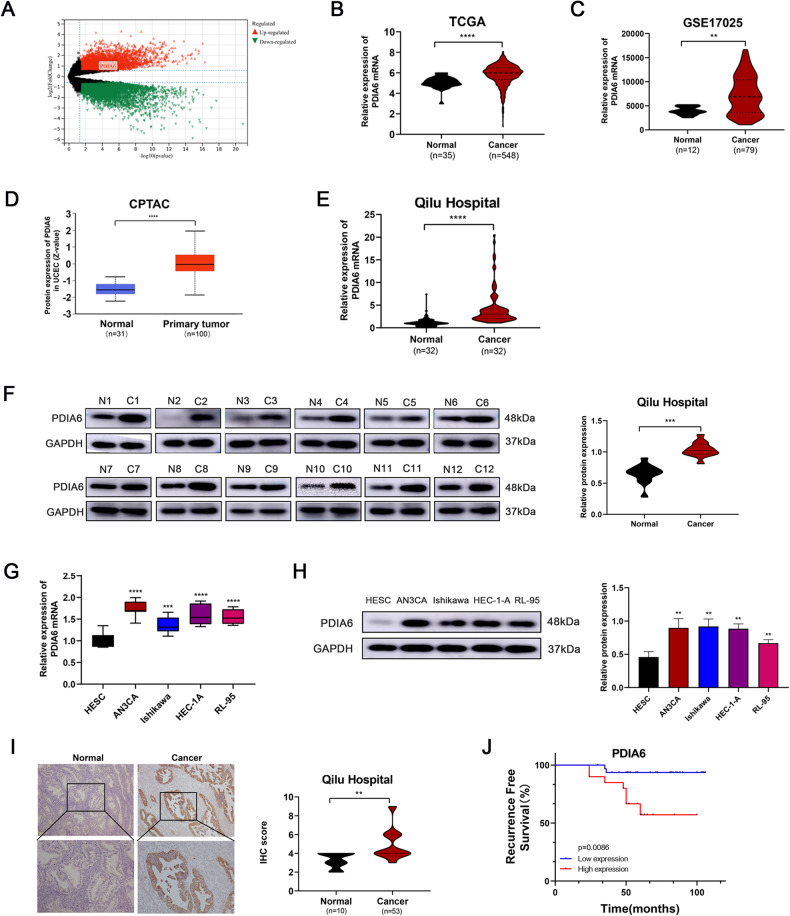
PDIA6 is upregulated and associated with the progression in endometrial cancer. **A** Volcano plots showing the expression profiles of differential genes in endometrial cancer (the red dots indicated high expression, and green dots indicated low expression). **B** The relative mRNA expression of PDIA6 in normal tissues and endometrial cancer tissues based on TCGA database. **C** The relative mRNA expression of PDIA6 in normal tissues and endometrial cancer tissues according to GSE17025. **D** PDIA6 protein expression analysis according to CPTAC database. **E** PDIA6 mRNA expression was higher in endometrial cancer tissues than normal tissues among the clinical samples which collected from Qilu Hospital. **F** PDIA6 protein expression and analysis in normal tissues and endometrial cancer tissues among the clinical samples (N1-N12, represented normal tissues; C1-C12, represented endometrial cancer tissues). **G** PDIA6 mRNA expression were higher in endometrial cancer cell lines(AN3CA, Ishikawa, HEC-1A and RL-95) than HESC. **H** PDIA6 protein expression were higher in endometrial cancer cell lines (AN3CA, Ishikawa, HEC-1A and RL-95) than HESC. **I** Representative IHC images and analysis of PDIA6 expression in normal tissues and endometrial cancer tissues. **J** Patients with higher PDIA6 expression had a shorter recurrence free survival than those with lower PDIA6 expression.

Volcano plot was used to show the differential genes between endometrial cancer tissues and normal tissues based on GSE17025 dataset. The red dots indicated the up-regulation and the green dots indicated the downregulation genes (Fig. 1A). According to TCGA database and GSE17025 dataset, the mRNA expression of PDIA6 in endometrial cancer tissues was higher than that in normal tissues (Fig. 1B, C). The protein expression of PDIA6 was increased in endometrial cancer tissues based on CPTAC database (Fig. 1D). Consistently, qRT-PCR was performed to verify the changes of PDIA6 expression in specimens pathologically diagnosed as endometrial cancer at Qilu Hospital, and the results showed that the mRNA level of PDIA6 in endometrial cancer tissues was frequently upregulated, compared to the normal tissues (Fig. 1E). PDIA6 protein levels verified by Western blot followed the same trend (Fig. 1F). At the same time, qRT-PCR results showed that PDIA6 mRNA were relatively highly expressed in endometrial cancer cell lines (Ishikawa, AN3CA, HEC-1A, RL-95) compared with HESC (Fig. 1G). PDIA6 protein expression levels were also upregulated in Ishikawa, AN3CA, HEC-1A and RL-95 compared with HESC, the differences were statistically significant (Fig. 1H). IHC results were carried to show that PDIA6 was highly expressed in endometrial cancer (Fig. 1I). In addition, the relationship between clinicopathological features and PDIA6 expression levels in patients with endometrial cancer was shown in Table I, indicating a significant correlation between PDIA6 expression and FIGO stage and lymph node metastasis in patients with endometrial cancer. Meanwhile, Kaplan–Meier survival analysis indicated that patients with higher PDIA6 expression had shorter recurrence free survival than those with lower PDIA6 expression (Fig. 1J). It is suggested that PDIA6 could be used as one of the indicators to predict the prognosis of patients with endometrial cancer.

**Table. I:** Association between PDIA6 expression and clinicopathologic characteristics of endometrial cancer patients.

Factors	Sample	PDIA6 expression Low High	*P*-value
Age			**0.5525**
≤50	16	9 7	
> 50	37	24 13	
Menopause			**0.3287**
NO	22	12 10	
Yes	31	21 10	
FIGO stage			0.0452*
I	39	28 11	
II	4	2 2	
III-IV	10	3 7	
Differentiation grade			**0.8697**
High+Middle	43	27 16	
Low	10	6 4	
Lymph node metastasis			0.0144*
Positive	6	1 5	
Negative	47	32 15	
Tumor size			**0.0942**
<5 cm	41	28 13	
≥5 cm	12	5 7	

### PDIA6 promotes endometrial cancer cell proliferation and metastasis

**Fig. 2:**
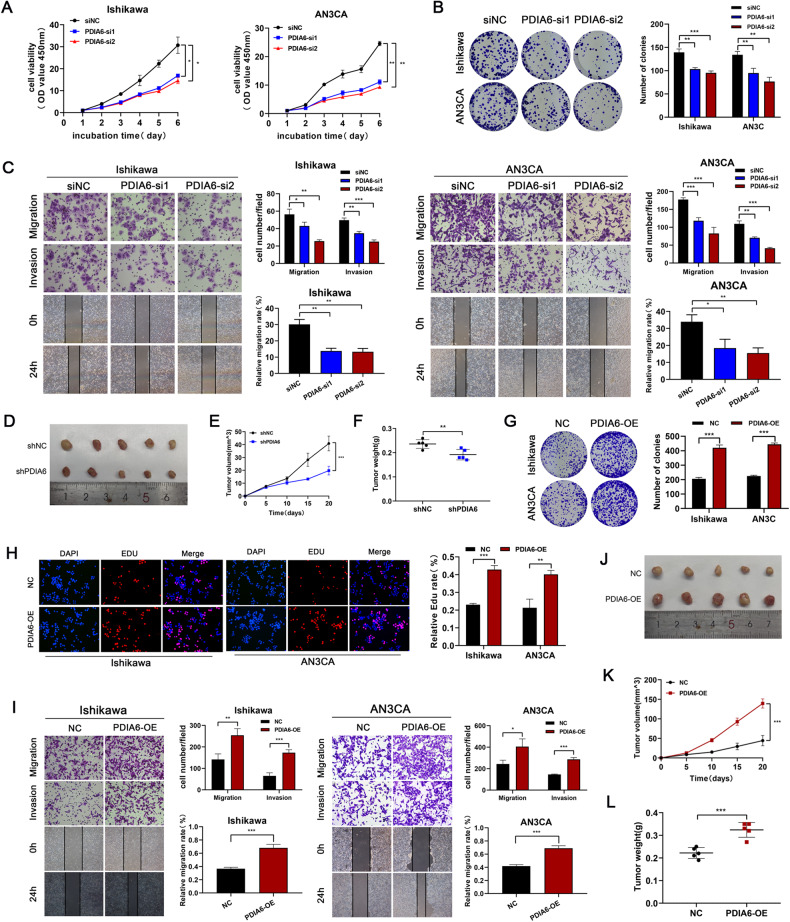
PDIA6 promotes cell proliferation and metastasis in endometrial cancer. **A** CCK8 assay was performed to detect the cell proliferation after PDIA6 knockdown in Ishikawa and AN3CA cells. **B** Colony formation assay proved that the proliferation of Ishikawa and AN3CA cells were inhibited after PDIA6 knockdown. **C** Transwell assay and wound healing assay confirmed that knockdown of PDIA6 in Ishikawa and AN3CA cells diminishes metastatic ability. **D** Representative images of xenograft tumors in the PDIA6 knockdown and control groups after subcutaneous injection of Ishikawa cells. **E** Tumor volumes was calculated after injection every 5 days and tumor growth curves were conducted. **F** Tumor weights were lower for xenograft tumors with PDIA6 knockdown than for xenograft tumors with control groups. **G** Cell viability was detected by colony formation assay while Ishikawa and AN3CA cells were transfected with NC and PDIA6-OE. **H** EDU assay were performed to identify proliferation after PDIA6 overexpressed in Ishikawa and AN3CA cells. **I** Metastatic ability in Ishikawa and AN3CA cells after PDIA6 overexpression were measured by Transwell assay and wound healing assay. **J** Representative pictures of PDIA6 overexpression group and control xenograft tumors after subcutaneous injection of Ishikawa cells. **K**, **L** Tumor volume and weight were higher for xenograft tumors with PDIA6 overexpression (PDIA6-OE) than controls (NC).

**Fig. 3:**
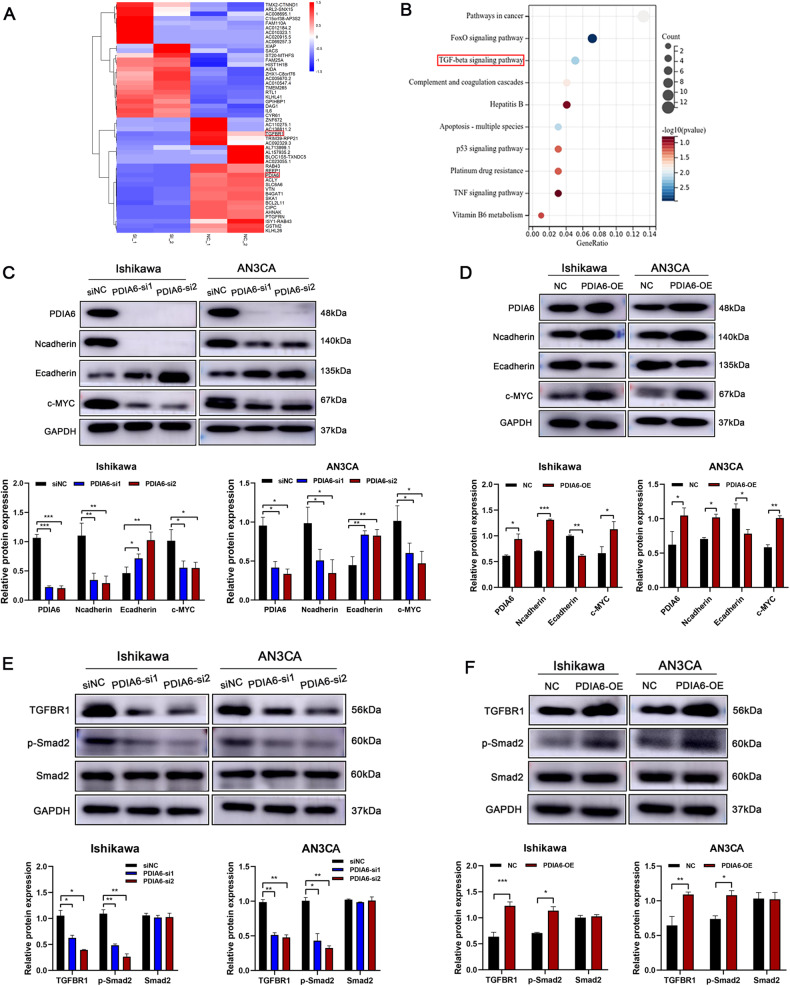
PDIA6 promotes the malignant phenotypes of endometrial cancer by regulating TGF-beta pathway. **A** Heat map of Top50 differentially expressed genes in PDIA6 knockdown cells compared to controls by transcriptome sequencing analysis. Red boxes indicated PDIA6 and TGFBR1. **B** KEGG pathway analysis was performed in PDIA6 knockdown cells compared to negative control cells. **C**, **D** Representative images and analysis of the protein expression of N-cadherin, E-cadherin and c-MYC detected by western blot in the control and PDIA6 knockdown groups. **E**, **F** Representative images and analysis of the protein expression of TGFBR1, Smad2 with its phosphorylated forms were evaluated by western blot in the control and PDIA6 knockdown groups.

Since the effects of PDIA6 in endometrial cancer has not been reported, we conducted a series of experiments to study the function of PDIA6 in endometrial cancer cells. Ishikawa and AN3CA cells were transfected with siRNAs against PDIA6, and qRT-PCR was used to verify the transfection efficiency. The results showed that PDIA6 expression decreased significantly in the PDIA6-si1/ PDIA6-si2 groups compared to the control group (Supplementary Fig. 1A). Then CCK8 assay showed that cell growth ability was inhibited significantly after PDIA6 knockdown (Fig. 2A), the colony formation assay showed the same trend (Fig. 2B). Transwell assay and Wound healing assay confirmed that knockdown PDIA6 could decrease the ability of migration and invasion in endometrial cancer cells compared with the control group respectively (Fig. 2C). To further elucidated the role of PDIA6 in endometrial cancer progression, we generated a subcutaneous xenograft tumor mouse model of endometrial cancer. Ishikawa cells with or without stable PDIA6 knockdown was subcutaneously injected into the armpit of female nude mice in two groups, and the tumor size was observed periodically for 4 weeks. As expected, PDIA6 knockdown reduced tumor size and weight (Fig. 3D–F).

On the other hand, after transfection of PDIA6 overexpressing lentivirus into endometrial cancer cells, qRT-PCR was used to verify the transfection efficiency. The results showed that PDIA6 expression increased significantly in the PDIA6-OE group compared to the control group (Supplementary Fig. 1B). The colony formation assay and the EDU assay suggested that the proliferation ability of endometrial cancer cells was increased compared with the control group (Fig. 2G, H). Transwell assay and Wound healing assay showed that the metastatic ability of endometrial cancer cells was significantly enhanced after PDIA6 expression was upregulated (Fig. 2I). Moreover, PDIA6 overexpression could increase the volume and weight of subcutaneous tumors in nude mice (Fig. 3J–L). In summary, these findings all together illustrated the carcinogenic effect of PDIA6 in the malignant biological behavior of endometrial cancer.

### PDIA6 could activate TGF-beta signaling in endometrial cancer cells

To investigate the molecular mechanism of PDIA6 in endometrial cancer progression in depth, we knocked down PDIA6 in AN3CA cells and performed transcriptome sequencing. Then, we analyzed the transcriptome sequencing results using a bioinformatics method. Heat map was used to show the Top50 differential genes (Fig. 3A). The Kyoto Encyclopedia of Genes and Genomes (KEGG) pathway enrichment analysis of all differential genes revealed that PDIA6 was associated with TGF-β signaling pathway closely (Fig. 3B). Therefore, we focused on TGFBR1, an important molecule in the TGF-β signaling pathway. Western blot analysis showed that PDIA6 knockdown decreased the protein expression of c-MYC, a marker associated with proliferation. PDIA6 knockdown elevated the protein expression of E-cadherin (an epithelial marker) and decreased the protein expression of N-cadherin (a mesenchymal marker), which were associated with migration and invasion. While PDIA6 overexpression exerted the opposite effect in Ishikawa and AN3CA cells (Fig. 3C, D). It is suggested that PDIA6 could affect the proliferation and metastatic ability of EC cells. More importantly, after confirming the alteration of TGFBR1 in EC cells by PDIA6 knockdown or overexpression, we found that the activation of the downstream signaling cascade of the TGF-β signaling pathway was subsequently altered (Fig. 3E, F). Therefore, PDIA6 could accentuate the progression of EC by regulated TGF-β signaling pathway.

### PDIA6 is regulated by TRPM2-AS/miR-424-5p axis in endometrial cancer

**Fig. 4:**
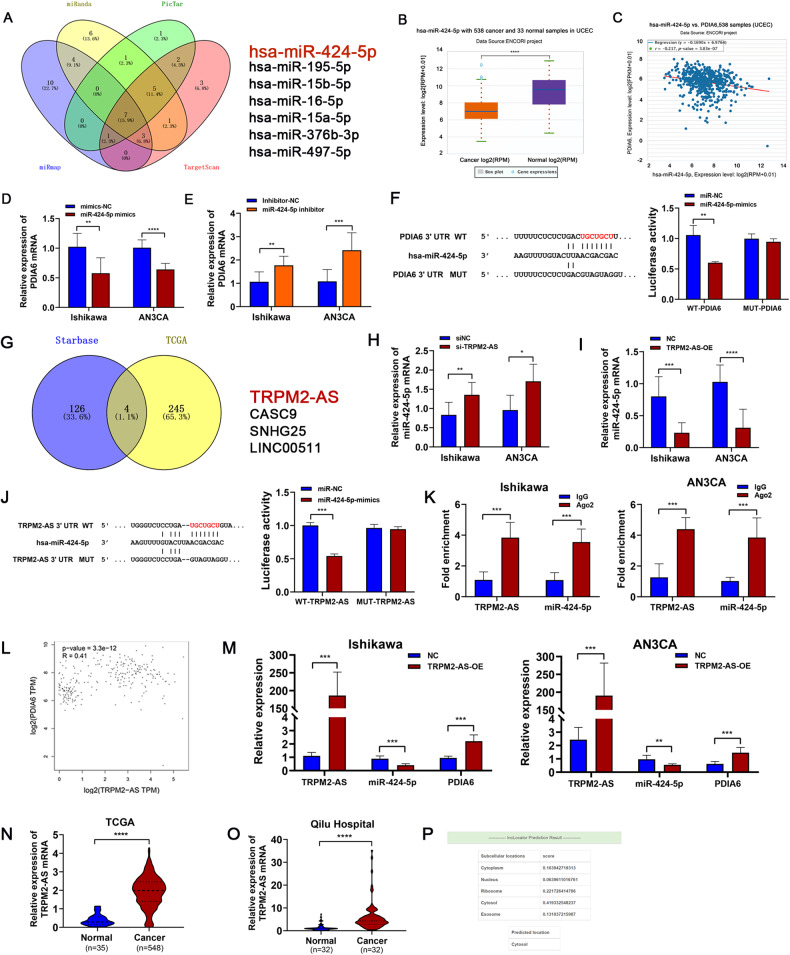
PDIA6 was regulated by TRPM2-AS /miR-424-5p axis. **A** Venn diagram displaying the upstream miRNA of PDIA6 predicted by miRanda, PicTar, miRmap and TargetScan. **B** The relative expression of miR-424-5p in endometrial cancer tissues and normal tissues based on the TCGA database. *p* = 1.90E-15. **C** The co-expression analysis for miR-424-5p and PDIA6 interactions from StarBase. **D**, **E** The expression of PDIA6 was decreased by the miR-424-5p mimics while the inhibitor made the opposite effect in Ishikawa and AN3CA cells. **F** Luciferase reporter assay was performed to detect the targeting relationship between miR-424-5p and PDIA6. **G** Schematic illustration showing the upstream lncRNAs of miR-424-5p predicted by the StarBase and TCGA database. **H**, **I** The relative expression of miR-424-5p was detected by qRT-PCR after TRPM2-AS knockdown (si-TRPM2-AS) or overexpression (TRPM2-AS-OE) in Ishikawa and AN3CA cells. **J** Luciferase reporter assay was performed to detect the targeting relationship between TRPM2-AS and miR-424-5p. **K** RIP experiments for TRPM2-AS and miR-424-5p were performed in Ishikawa and AN3CA cells, TRPM2-AS and miR-424-5p were more enriched by Ago2 than IgG. **L** Analysis of the correlation between TRPM2-AS and PDIA6 using the TCGA database. **M** Overexpressed TRPM2-AS could reduce miR-424-5p expression and increased PDIA6 expression in Ishikawa and AN3CA cells, **N** Column chart showing the mRNA expression of TRPM2-AS in normal tissues and endometrial cancer tissues. **O** qRT-PCR analysis was used to detect the expression of TRPM2-AS in normal tissues and endometrial cancer tissues among the clinical samples which collected from Qilu Hospital. **P** The location of TRPM2-AS was predicted by lncLocater (long non-coding RNA subcellular localization predictor) website.

To investigate the potential molecular mechanism of PDIA6 promotion in tumors, we investigated whether non-coding RNAs could regulate PDIA6 levels in EC patients. MiRanda: http://www.miranda.org/, miRmap: https://mirmap.ezlab.org/, PicTar: https://pictar.mdc-berlin.de/ and Targetscan7.2: https://www.targetscan.org/vert_72/ were intersected to identify the potential upstream regulators of PDIA6. According to the joint prediction of multiple databases, we found that miR-424-5p, miR-195-5p, miR-497-5p, miR-15b-5p, miR-16-5p, miR-15a-5p and miR-376b-3p contains potential binding sites with PDIA6 (Fig. 4A). Next, according to the miRNA-mRNA regulatory mechanism, we selected the intersection of the above miRNAs that were upregulated in both the TCGA database and GSE25405 dataset. Finally, three miRNAs meeting these requirements were chose, included miR-424-5p (Fig. 4B), miR-195-5p and miR-497-5p (Supplementary Fig. 1C). Moreover, the expression levels of miR-424-5p and PDIA6 in endometrial cancer samples and their relationship were analyzed based on the ENCORI project (Fig. 4C, *r* = −0.217, *p* = 3.83e-07). Then qRT-PCR was used to detect the expression of PDIA6 after transfection of the mimics of the three microRNA. The results showed that only miR-424-5p mimics decreased PDIA6 expression (Fig. 4D), miR-195-5p and miR-497-5p could not alter PDIA6 expression in endometrial cancer cells (Supplementary Fig. 1D, E). Meanwhile, we transfected miR-424-5p inhibitor in EC cells and PDIA6 expression was subsequently increased (Fig. 4E). In addition, we constructed plasmids carrying wild-type and mutant PDIA6 sequences for the dual luciferase reporter assay. 293 T cells were co-transfected with luciferase plasmid and miR-424-5p mimics, and fluorescence was detected after 36 h. Notably, miR-424-5p reduced the luciferase activity of PDIA6 wild-type, but there was no change in the luciferase activity of the mutant (Fig. 4F), which indicated that miR-424-5p was the upstream regulator of PDIA6.

Long non-coding RNAs (LncRNAs) are RNA molecules with transcript length of over 200 bp but are not translated into proteins [9]. Recently, plenty of lncRNA have been reported to play pivotal roles in tumor progression though the competing endogenous RNAs (ceRNAs) mechanism [10]. We selected the potential lncRNAs that may interact with miR-424-5p by the online tool Starbase (https://starbase.sysu.edu.cn/). Then intersected with different lncRNAs which were upregulated in endometrial cancer according to TCGA database (logFC > 1.5). In the end, four lncRNAs that meet these requirements were finally screened, TRPM2-AS, CASC9, SNHG25, LINC00511 (Fig. 4G). To further prove this prediction, qRT-PCR was conducted to detect the miR-424-5p expression after their siRNA transfection. We found that only TRPM2-AS could regulate miR-424-5p expression in both Ishikawa and AN3CA cell lines (Fig. 4H, I), while CASC9, SNHG25 and LINC00511 could not (Supplementary Fig. 1F–H). Furthermore, dual luciferase reporter assay was carried in 293 T cell. The results showed that after co-transfecting TRPM2-AS-wild type and miR-424-5p mimics, the fluorescence intensity was reduced; however, group of co-transfecting TRPM2-AS-mutant type and miR-424-5p mimics had no differences (Fig. 4J). As expected, the RIP assay revealed that TRPM2-AS and miR-424-5p were enriched in the Ago2-containing immunoprecipitants from Ishikawa and AN3CA cells compared with the control IgG groups (Fig. 4K). These results indicated that TRPM2-AS could directly bind miR-424-5p and may serve as a ceRNA for miR-424-5p in endometrial cancer cells. Meanwhile, bioinformatics analysis showed the expression of PDIA6 was positively correlated with TRPM2-AS (Fig. 4L). Then, qRT-PCR was performed to show that overexpressed TRPM2-AS reduced the expression of miR-424-5p but increased the expression of PDIA6 in Ishikawa and AN3CA cell lines (Fig. 4M).

TRPM2-AS, an antisense lncRNA of TRPM2 locating at chromosome 21q22.3 [11], was first found to be upregulated in melanoma in 2008 [12]. TRPM2-AS has been reported as an oncogene that sponges miRNAs in different tumors [10], such as bladder cancer [13], gastric cancer [14], ovarian cancer [15] and lung cancer [10]. TRPM2-AS has been shown to be a tumor promoter in bladder cancer [13], ovarian cancer [15] and gastric cancer [16] through its sponge for miRNA. To determine the possible involvement of TRPM2-AS in endometrial cancer, we analyzed the TCGA database. The results showed that TRPM2-AS was overexpressed in EC tissues than in normal tissues (Fig. 4N). We validated the results in 32 pairs of endometrial cancer tissues and normal tissues from patients with pathologically diagnosed endometrial cancer at Qilu Hospital of Shandong University, and the results were the same as before (Fig. 4O). Moreover, TRPM2-AS was mainly located in the cytoplasm predicated by lncLocater (long non-coding RNA subcellular localization predictor) website (http://www.csbio.sjtu.edu.cn/bioinf/lncLocator/) (Fig. 4P). These results indicated that TRPM2-AS may be correlated with endometrial cancer progression.

### TRPM2-AS promotes cell proliferation, migration and invasion in endometrial cancer

**Fig. 5:**
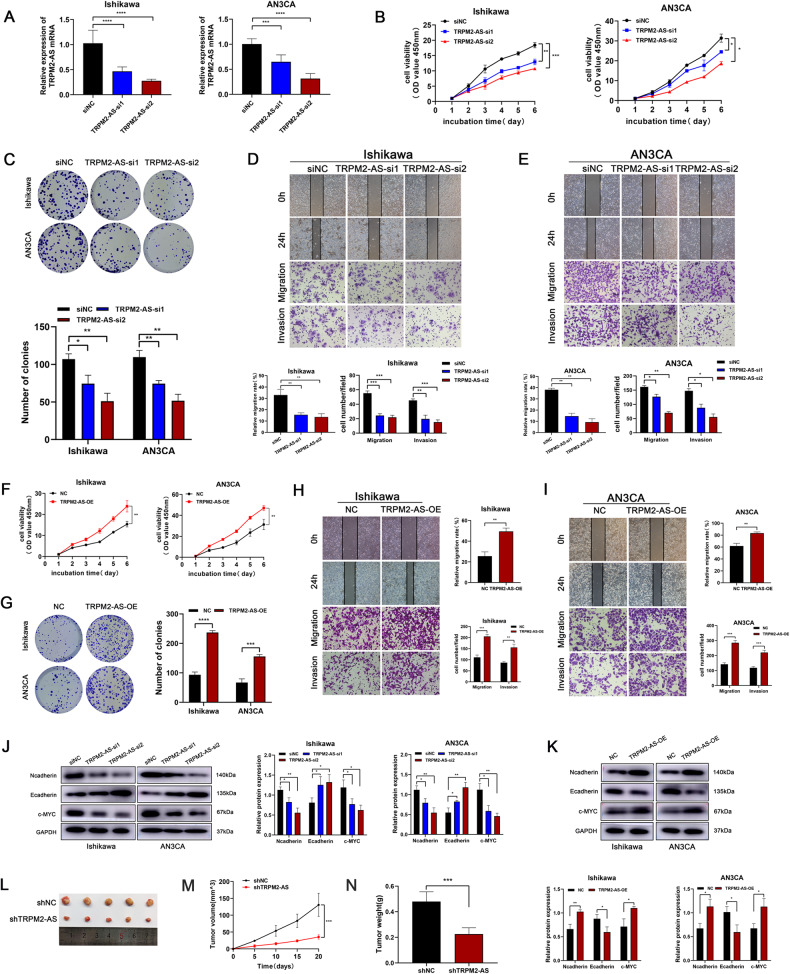
TRPM2-AS promotes cell proliferation and metastasis in endometrial cancer. **A** The transfection efficiency of TRPM2-AS transfected of siNC or si-TRPM2-AS were determined by qRT-PCR. **B**, **C** Cell proliferation were detected by CCK8 assay and colony formation assay after the knockdown of TRPM2-AS in Ishikawa and AN3CA cells. **D**, **E** Motility of Ishikawa cells and AN3CA cells transfected with siNC and si-TRPM2-AS were examined by wound healing assay and Transwell assay. **F** CCK8 assay indicated an increased proliferative ability of Ishikawa and AN3CA cells after TRPM2-AS overexpression. **G** Colony formation assay proved that the proliferation of Ishikawa and AN3CA cells were promoted after TRPM2-AS overexpressed. **H**, **I** Wound healing assay and Transwell assay indicated an increased migration and invasion ability of Ishikawa and AN3CA cells after TRPM2-AS overexpression. **J**, **K** Western blot detection of N-cadherin, E-cadherin and c-MYC proteins after TRPM2-AS knockdown or overexpression in Ishikawa and AN3CA cells. **L** Representative tumor images of TRPM2-AS knockdown (shTRPM2-AS) group and control group after subcutaneous injection with Ishikawa cells. **M**, **N** Tumor volume and weight of xenograft tumors were significantly decreased in the TRPM2-AS knockdown groups than control groups.

To investigate the effects of TRPM2-AS in endometrial cancer cells, Ishikawa and AN3CA cell lines were transfected with siRNAs against TRPM2-AS. The transfection efficiency was tested by qRT-PCR and the results showed that the expression levels of TRPM2-AS decreased significantly in the knockdown groups compared to the control group (Fig. 5A). After silencing TRPM2-AS, we performed cell function assays to study its role in endometrial cancer cells. The growth curves detected by CCK8 assay showed that TRPM2-AS knockdown significantly inhibited the growth of endometrial cancer cells (Fig. 5B). Moreover, colony formation assay displayed the same effect of silencing TRPM2-AS as presented in Fig. 5C. There is no doubt that these data verified the essential role of TRPM2-AS in regulating endometrial cancer cells proliferation. Metastasis is a prominent feature of endometrial cancer, so we further elucidated the functional significance of TRPM2-AS in the migration and invasion of endometrial cancer cells. We examined the effect of silencing TRPM2-AS on the metastatic activity of endometrial cancer cells by the wound healing assay, the results showed that depleting TRPM2-AS impaired endometrial cancer cell migration. Then Transwell assay was performed, the results showed that the number of cells passing through the chamber was lower in the knockdown groups than the control group (Fig. 5D, E). On the other hand, when EC cells were transfected with overexpressing lentivirus containing TRPM2-AS sequence, CCK8 and colony formation assay indicated that TRPM2-AS overexpression promoted the proliferation of endometrial cancer cells (Fig. 5F, G). Wound healing assay and Transwell assay displayed a significant increase in EC cell metastasis after TRPM2-AS overexpression (Fig. 5H, I).

We further evaluated the effect of TRPM2-AS on proliferation and metastasis-related markers. Western blot analysis showed that TRPM2-AS knockdown increased the expression of epithelial marker (E-cadherin) but decreased the expression of mesenchymal marker (N-cadherin) and c-MYC (Fig. 5J). Conversely, the expression of related proteins showed an opposite trend after TRPM2-AS overexpressed (Fig. 5K). These results all together indicated that the high expression of TRPM2-AS could promote endometrial cancer cell tumorigenesis and metastasis. Nude mice transplanted tumor model was performed to verify the reliability of the biologic effect of TRPM2-AS in vivo. The treated cells were injected subcutaneously into nude mice. It was obvious that knockdown TRPM2-AS could reduce the tumor size (Fig. 5L). The tumor volume growth curve showed that the tumors formed by control cells grew faster than the treated cells after inoculation (Fig. 5M), and the same trend was drawn from the tumor weight (Fig. 5N).

### Inhibition of miR-424-5p reversed the suppressive effects of TRPM2-AS knockdown on the malignant phenotypes of endometrial cancer cells

**Fig. 6:**
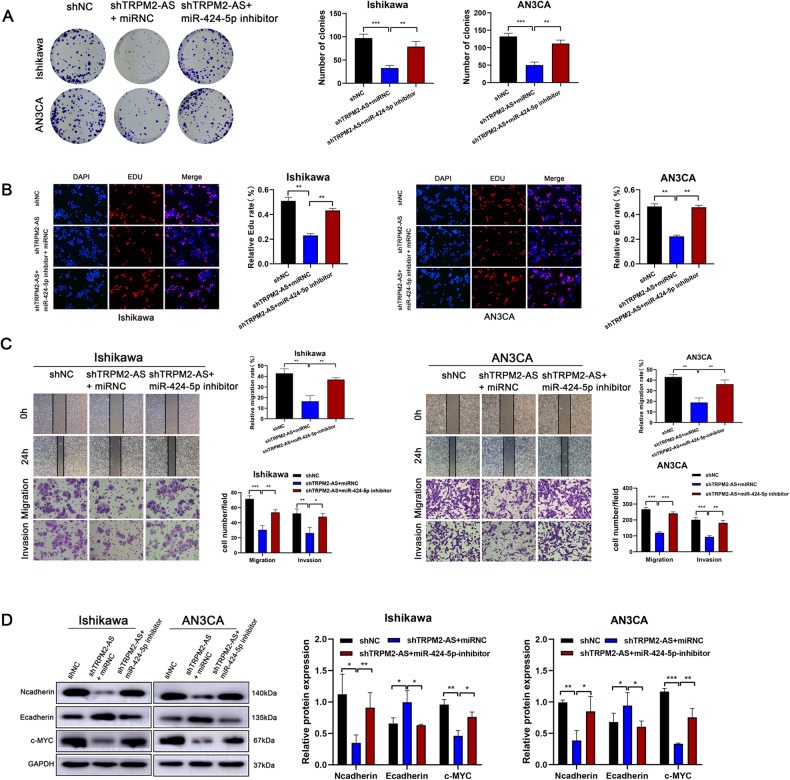
MiR-424-5p inhibitor diminished TRPM2-AS knockdown on endometrial cancer malignant phenotypes. **A**, **B** Colony formation assay and EDU assay were carried to investigate the effect of miR-424-5p inhibitor on cell viability caused by TRPM2-AS downregulation. **C** Wound healing and Transwell assays were performed to examine the effects of miR-424-5p inhibitor on cell migration and invasion induced by downregulation of TRPM2-AS. **D** Different expression and analysis of the protein levels of N-cadherin, E-cadherin and c‐MYC were examined by western blotting assay following transfection with shTRPM2-AS and miR-424-5p inhibitor.

To verify whether TRPM2-AS affected endometrial cancer cells by regulating miR-424-5p, Ishikawa and AN3CA cells were co-transfected with the shRNA against TRPM2-AS and miR-424-5p inhibitor. The colony formation assay indicated that the inhibition of miR-424-5p could reverse the significant inhibition of cell proliferation caused by TRPM2-AS knockdown (Fig. 6A). The EDU experiment showed the same trend (Fig. 6B). Similarly, the results of the Wound healing and Transwell assays indicated that the inhibition of miR-424-5p attenuated the suppressive effect of TRPM2-AS knockdown on cell migration and invasion in Ishikawa and AN3CA cells (Fig. 6C). As shown in Fig. 6D, miR-424-5p inhibitor could reverse the effect of TRPM2-AS knockdown on the expression of N-cadherin, E-cadherin and c-MYC in Ishikawa and AN3CA cells. Taken together, the rescue experiments further validated the functional relationship between TRPM2-AS and miR-424-5p, which was that TRPM2-AS promoted the malignant behavior of endometrial cancer cells by regulating miR-424-5p expression.

### PDIA6 overexpression rescued TRPM2-AS knockdown on endometrial cancer malignant phenotypes

**Fig. 7:**
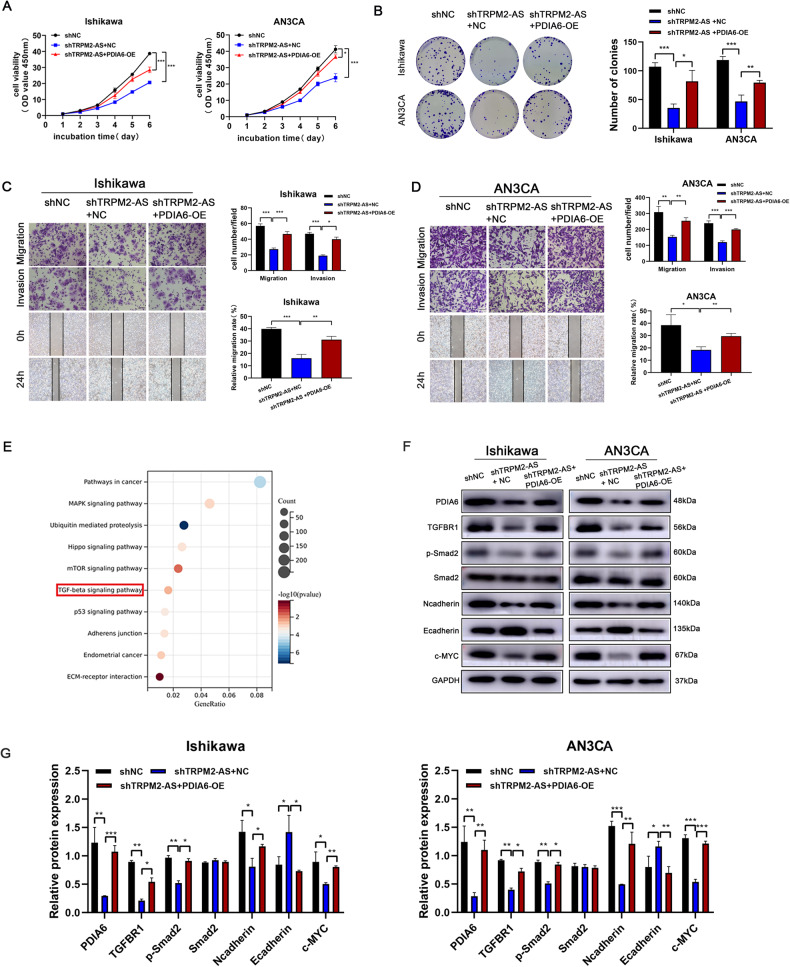
PDIA6 overexpression rescued TRPM2-AS knockdown on endometrial cancer malignant phenotypes. **A**, **B** Cell viability were detected by CCK8 and colony formation assays while Ishikawa and AN3CA cells were transfected with shTRPM2-AS and PDIA6-OE. **C**, **D** Cell motility were tested by Transwell and wound healing assays while Ishikawa and AN3CA cells were transfected with shTRPM2-AS and PDIA6-OE. **E** KEGG pathway analysis was performed in TRPM2-AS knockdown and control groups based on GSE40687. **F**, **G** Protein levels and analysis of PDIA6, TGFBR1, p-Smad2, Smad2, N-cadherin, E-cadherin and c‐MYC expression under different treatments in Ishikawa and AN3CA cells.

To further verify whether PDIA6 upregulation could attenuate the effect of TRPM2-AS knockdown in endometrial cancer cells, rescue assay was carried. We co-transfected TRPM2-AS knockdown and PDIA6 overexpression lentiviruses into endometrial cancer cells. The results of CCK8 and colony formation assays showed that PDIA6 overexpression counteracted the inhibitory effect of TRPM2-AS knockdown on proliferation in Ishikawa and AN3CA cells (Fig. 7A, B). Transwell and wound healing assays indicated that PDIA6 overexpression counteracted the inhibitory effect of TRPM2-AS knockdown on migration and invasion in Ishikawa and AN3CA cells (Fig. 7C, D). In parallel, we analyzed data from the GSE40687 dataset and used KEGG pathway enrichment analysis to show that TRPM2-AS was also closely related to the TGF-β signaling pathway (Fig. 7E). Western blot analysis showed that overexpression of PDIA6 counteracted the effect of TRPM2-AS downregulation on the expression of TGFBR1, p-Smad2, N-cadherin, E-cadherin and c-MYC (Fig. 7F, G). Thus, all these results described above indicated that PDIA6 was downstream effector of TRPM2-AS and could promote proliferation and metastasis process by regulating TGF-β signaling pathway in endometrial cancer.

## Discussion

Endometrial cancer is one of the most common gynecological malignancies in the world, and accounts for 7% of female cancers. It ranks fourth among the most common cancers in women, and the incidence rate is still increasing in recent years [17]. Moreover, endometrial cancer is considered to be a highly heterogeneous disease characterized by different tissue types and multiple genetic changes [4, 18]. Due to the molecular diversity in endometrial cancer patients, it may lead to the risk of undertreatment or overtreatment [4].

In this research, we found PDIA6 was overexpressed in endometrial cancer, and PDIA6 could regulate the proliferation and metastatic ability of endometrial cancer cells. Therefore, we indicated that PDIA6 may be an oncogene and play an essential role in promoting endometrial cancer progression. Further study of transcriptome sequencing results revealed that PDIA6 expression was closely associated with the TGF-β signaling pathway. The transforming growth factor-β (TGF-β) signaling pathway has an important role in regulating cell proliferation, apoptosis, metastasis, angiogenesis and EMT process in a variety of cancers [19, 20], including prostate cancer [21], lung cancer [22] and breast cancer [23]. And the TGF-β signaling pathway exerts multiple physiological roles in vivo through two main pathways: the classical SMAD-dependent pathway and the non-SMAD-dependent pathway [24–26]. TGFBR1 encodes the TGF-beta receptor 1 subunit of the receptor complex and plays an important role in the classical SMAD-dependent pathway and the non-SMAD-dependent pathway [27]. In our study, we found that PDIA6 regulated TGFBR1 expression in endometrial cancer, which in turn lead to attenuated activation of the downstream cascade of the TGF-β signaling pathway, suggested that PDIA6 promoted cell proliferation, migration and invasion by regulating the TGF-β signaling pathway through a classical SMAD-dependent pathway

It is well known that the ceRNA hypothesis has been broadly researched, and an expanding number of researches have investigated the effect of the complex network mechanism of the lncRNA/miRNA/mRNA axis in a wide range of human cancers [28, 29]. As a research hotspot, lncRNA could regulate cell biological processes through a variety of different molecular mechanisms, including roles in regulating gene expression, imprinting, transcription, and post-translational processing [30, 31], making the process of gene regulation extremely complicated [32]. Despite the functional diversity of lncRNA, there is no doubt that they are involved in the occurrence and development of various cancers [9, 33, 34]. Recent studies have shown that there are a large number of lncRNA expression disorders in endometrial cancer, and their abnormal expression is related to tumor occurrence, metastasis, prognosis and diagnosis [9, 28, 35]. Among them, TRPM2-AS have been found to be abnormally expressed in ovarian cancer [15], gastric cancer [16] and osteosarcoma [36] et al. MicroRNAs (miRNAs), as primary elements of non-coding RNAs, have a substantial influence in multiple biological processes such as cell proliferation, apoptosis, metastasis, tumorigenesis and cancer regulation by interacting with lncRNA [37–41]. In the present study, we found that PDIA6 could be regulated by the TRPM2-AS/miR-424-5p axis to promote the proliferation and metastasis process of EC cells. In addition, we determined that high TRPM2-AS expression could promote the proliferation and metastatic ability of endometrial cancer cells. The subsequent rescue experiments showed that the inhibitory impacts of TRPM2-AS knockdown on growth and metastasis of endometrial cancer, were attenuated by miR-424-5p downregulation or PDIA6 overexpression both in phenotype and protein molecular level. Therefore, we demonstrated that PDIA6 expression was regulated by TRPM2-AS/miR-424-5p axis in endometrial cancer.

## Conclusion

In conclusion, this study proved that PDIA6 is upregulated in endometrial cancer tissues and high PDIA6 expression is associated with tumor progression. We demonstrated that PDIA6 promotes endometrial cancer cell proliferation and metastasis through TGF-β signaling pathway for the first time. Furthermore, TRPM2-AS/miR-424-5p axis was verified as the upstream molecule that directly targeted PDIA6 and regulated the biological function of PDIA6 in endometrial cancer. Therefore, our research may help reveal an effective therapeutic strategy for endometrial cancer patients. We believed that with the rapid development of scientific research and the continuous updating of technology, more and more therapeutic targets and new biomarkers for endometrial cancer could be developed.

### Supplementary information

Supplementary Materials

Supplementary Table.I

Supplymentary Figure 1

Original Data File

A change of the manuscript

aj-checklist

## Data Availability

The data used or analyzed in this study are available from the corresponding authors on justified demand.
