# Supplementary material for: PDIA6, which is regulated by TRPM2-AS/miR-424-5p axis, promotes endometrial cancer progression via TGF-beta pathway
Source: Cell Death Dis. 2023 Dec 14;14(12):829. doi: 10.1038/s41419-023-06297-8 (PMC10721792; doi:10.1038/s41419-023-06297-8)
Supplement: Supplementary file 1 — Supplementary Materials [file 41419_2023_6297_MOESM1_ESM.docx]

**Supplementary Materials and methods**

**2.2. Cell culture**

Endometrial cancer cell lines (Ishikawa and AN3CA) were obtained from the Key Laboratory of Gynecologic Oncology of Shandong Province which were tested and identified. Ishikawa cells were cultured in RPMI 1640 medium supplemented with 10% fetal bovine serum (FBS) and 1% antibiotics (penicillin and streptomycin) and incubated in a sterile incubator at 37°C. AN3CA cells were cultured in DMEM/F12 medium in the same treatment.

**2.3. SiRNA and lentivirus transfection**

For transient transfection, small interfering RNA and control small interfering RNA (siNC) were purchased from GenePharma (Shanghai, China). MicroRNA mimics, inhibitor and corresponding controls (miRNC) were purchased from GenePharma (Shanghai, China). INTERFERin (Polyplus, Shanghai, China) was used to transient transfection according to manufacturer’s instructions. For stable transfection, lentiviruses carrying target gene knockdown sequence or corresponding controls (shNC), target gene overexpression sequence or corresponding controls (NC) were purchased from Genechem (Shanghai, China). Then transfected into endometrial cancer cells according to manufacturer’s instructions and selected with puromycin for about 7 days. All sequences were listed in Supplementary Table.I.

**2.4. RNA Extraction and Quantitative Real-Time PCR**

Total RNA was extracted from cell and tissue samples using TRIzol reagent (Invitrogen; CA, USA) and reverse transcribed using ReverTra Ace qPCR RT Master Mix with gDNA Remover (Code FSQ-301, Toyobo, Japan) according to the manufacturer's instructions. RNA expression was quantified by qRT-PCR using 2x SYBR Green qPCR Mix (SparkJade, China). GAPDH and U6 were used as internal controls for mRNA and miRNA respectively. The relative expression of mRNA was calculated by the 2^-ΔΔCt^ method. The primer sequences used in this research are listed in Supplementary Table.I.

**2.5. Western blotting**

Cells were washed 3 times with PBS and fully lysed using RIPA cell lysis buffer (Beyotime; China) containing 1% phenylmethylsulfonyl fluoride (PMSF) and 1% NaF for 30 min on ice. Samples were centrifuge at 12,000g for 15 min at 4°C and collected the supernatant. Protein concentration was measured by BCA protein assay kit (Merck Millipore, USA). Then boiled in a 100° metal bath for 10 min, and stored in -20 refrigerator. 30-50μg of each sample was taken for protein separation by sodium dodecyl sulfate-polyacrylamide gel electrophoresis and the protein was transferred to a polyvinylidene fluoride membrane (Merck Millipore, Burlington, MA, USA). After being closed with 5% milk powder for 2h at room temperature, the membranes were left overnight at 4 °C incubated with the target primary antibody PDIA6(1:500; Santa Cruz biotechnology; sc365260; mouse), TGFBR1(1:1000; ABclonal;A22151;rabbit),p-Smad2(1:1000;ABclonal; AP0269; rabbit),Smad2(1:1000; ABclonal;A7699;rabbit), N-cadherin (1:1000; Cell Signaling Technology; #13116; rabbit), E-cadherin(1:1000;Cell Signaling Technology; #14472; rabbit), c-MYC (1:1000; Proteintech;10828-1-AP; rabbit) and GAPDH (1:2000; Servicebio; GB11002; rabbit). The following day, after washing 3 times with TBST, the membrane was incubated with secondary antibody for 1h at room temperature. The signal was then detected by using the M5 Hiper ECL Western HRP Substrate (Cat.MF074-05, China) according to the manufacturer's recommendations.

**2.6. Immunohistochemistry (IHC)**

Paraffin-embedded tissues were cut into 4-μm sections. Sections were dried, dewaxed, hydrated, and microwave antigen repaired, and then endogenous peroxidase activity was blocked with 3% hydrogen peroxide for 15min at room temperature. The slides were closed with normal goat serum in a humidified chamber at 37°C for 30 min. Sections are then incubated overnight at 4°C with primary antibody PDIA6 at a dilution of 1:100. The next day, the sections were incubated with horseradish enzyme labeled Streptomyces ovalbumin working solution (Zhongshan Golden Bridge, SP-9000) for 1h at room temperature. Finally, the sections were tested for signals with 3,3' -diaminobenzidine (DAB) chromogenic agent and then counterstained with hematoxylin. IHC results were evaluated independently by two experienced pathologists. A score value less than 6 was defined as PDIA6 low expression, while a staining index greater than or equal to 6 was defined as PDIA6 high expression.

**2.7.** **Luciferase reporter assay**

The wild-type and mutant 3ʹ UTRs of TRPM2-AS and PDIA6 were cloned into the pmirGLO vector (Promega, USA). Then miR-424-5p mimics or control mimics were co-transfected into 293T cells with the indicated reporter plasmids using EndoFectin ^TM^ Max Transfection Reagent (Cat. No: EF013, China). After 48 hours, the Dual-Luciferase Reporter Gene Assay Kit (Beyotime, China) was used to analyze relative luciferase activity according to the manufacturer's protocol.

**2.8. RNA immunoprecipitation (RIP) assay**

RIP assays were performed by using the EZ-Magna RIP RNA-binding Protein Immunoprecipitation Kit (Millipore, Burlington, MA, USA) to verify the target relationship between lncRNA-TRPM2-AS and miR-424-5p according to the manufacturer’s protocol. Endometrial cancer cells were collected and lysed with RIP lysis buffer on ice for 5 min and stored at -80 °C for later use. 50ul protein A/G beads suspension was washed, and added 5µg anti-Ago2 antibody (Cell Signaling Technology, #2897, rabbit) or 5µg IgG (Millipore, #PP64B, MA, USA) respectively, then rotated and incubated at room temperature for 30 min. Washed the protein A/G beads binding complex, and added the thawed cell lysate, then rotated and incubated at 4 °C overnight. 400µL phenol: chloroform: isopentyl alcohol=125:24:1 was added to each centrifuge tube to separate the immunoprecipitated RNA. The RNA was purified and reverse-transcribed into cDNAs for qRT-PCR.

**2.9. CCK8 assay**

Cell viability was assayed using the Cell Counting Kit-8 (CCK8) (APExBIO, #K1018) following the manufacturer's protocol. The treated cells were inoculated in 96-well plates and incubated overnight. 24, 48, 72, 96, 120, 144h later, 10μl CCK8 solution was added to each well and incubated at 37°C for 1 h. The absorbance of each well was measured at 450 nm by a microplate reader.

**2.10. Colony Formation Analysis**

Treated cells were seeded in 12-well plates and colony formation was observed after 14 days. Then, cells were fixed with methanol for 20 min and stained with 0.1% crystal violet solution for 30 min. Colony formation images were taken with a light microscope and the number of colonies was counted using ImageJ software.

**2.11. Transwell assay**

Treated cells were seeded into 200 µl serum-free medium and in the top Boyden chambers (8μm pores, BD Biosciences, USA) which were inserted into 24-well plates without or with Matrigel (BD Biosciences, USA), and complete medium (700 µL) with 20% FBS was placed in the lower chamber. After incubated at 37 ℃ for 24-48 hours, the migrated or invaded cells on the lower surface were fixed with methanol for 20 min and stained with 0.5% crystal violet for 20 min. Then a light microscope was used to observe and count the migrated or invaded cells.

**2.12.** **Wound healing assay**

Treated cells were seeded at the density of 1×10^6 cells/well into 6-well plates and incubated at 37 ℃ for a night. 100ul sterile pipette tips was used to make straight scratches until the cells reached 90% confluence. Then the cells were cultured in fresh medium and incubated at 37 ℃ for 24 hours. 0 and 24 hours were taken images by an inverted fluorescence microscope at the same location. Mobility was calculated as the ratio of the difference between the 0h and 24h gap distances to the 0h gap distance.

**2.13. Animal experiment**

Animal experiments were approved by the Experimental Animal Ethics and Welfare Committee of Qilu Medical College, Shandong University. Female nude mice aged 4-6 weeks were randomly divided into 4 groups. Stable knockout or overexpression target genes and their respective corresponding controls were constructed for endometrial cancer cells and injected into the axillary skin of nude mice. Tumor size was measured weekly for 20 days. The tumor volume was calculated as length (mm) × width^2 (mm^2) × 0.5. After 20 days, the tumors of mice were excised and weighed.

**2.14. Statistical analysis**

At least three independent replicates of each test were performed. Data satisfied normal distribution. Statistical comparison between two and more than two groups was performed using Student’s t test or One-way ANOVA. Image processing was performed using GraphPad Prism 8.01 and photoshopcs6 (USA). (**p < 0.05, **p < 0.01, ***p < 0.001*).

**Supplementary Fig.1**

1. The mRNA expression of PDIA6 was examined by qRT-PCR after transfection of siNC and si-PDIA6 in Ishikawa and AN3CA cells; B. The mRNA expression of PDIA6 was examined by qRT-PCR after transfection of NC and PDIA6-OE in Ishikawa and AN3CA cells; C. The relative expression of miR-195-5p and miR-497-5p in endometrial cancer tissues and normal tissues based on the TCGA database; D-E. The mRNA expression of PDIA6 was detected by qRT-PCR after miR-195-5p mimics and miR-497-5p mimics were transfected in Ishikawa and AN3CA cells; F-H. The relative expression of miR-424-5p was detected by qRT-PCR after CASC9, SNHG25 and LINC00511 were knockdown in Ishikawa and AN3CA cells.

**Supplementary Table.I**

All sequences of genes we used in this study were listed in Supplementary Table.I.
