# Supplementary material for: PDIA6, which is regulated by TRPM2-AS/miR-424-5p axis, promotes endometrial cancer progression via TGF-beta pathway
Source: Cell Death Dis. 2023 Dec 14;14(12):829. doi: 10.1038/s41419-023-06297-8 (PMC10721792; doi:10.1038/s41419-023-06297-8)
Supplement: Supplementary file 2 — Supplementary Table.I [file 41419_2023_6297_MOESM2_ESM.docx]

**Supplementary Table.I All the sequences of genes we used in this study**

| **siRNAs/miRNA Oligo/shRNA/gene** | | **Sequence (5’-3’)** |
| --- | --- | --- |
| PDIA6-si1 | Sense | GGCAGUGAAUGGUCUGUAUTT |
|  | Antisense | AUACAGACCAUUCACUGCCTT |
| PDIA6-si2 | Sense | GUGGCUUGUAGAAUUCUAUTT |
|  | Antisense | AUAGAAUUCUACAAGCCACTT |
| TRPM2-AS-si1 | Sense | CUCUGCAGUGCGGUAUGUGTT |
|  | Antisense | CACAUACCGCACUGCAGAGTT |
| TRPM2-AS-si2 | Sense | GAACCUUCCCUAAUAGAAATT |
|  | Antisense | UUUCUAUUAGGGAAGGUUCTT |
| CASC9-si | Sense | GCAGUCAUGUAAACUAUAATT |
|  | Antisense | UUAUAGUUUACAUGACUGCTT |
| SNHG25-si | Sense | CCAUUCCCGUCAAUAAAGUTT |
|  | Antisense | ACUUUAUUGACGGGAAUGGTT |
| LINC00511-si | Sense | CUCCCUUGAACUGCUAGAUTT |
|  | Antisense | AUCUAGCAGUUCAAGGGAGTT |
| Negative control | Sense | UUCUCCGAACGUGUCACGUTT |
|  | Antisense | ACGUGACACGUUCGGAGAATT |
| mir-424-5p mimics | Sense | CAGCAGCAAUUCAUGUUUUGAA |
|  | Antisense | CAAAACAUGAAUUGCUGCUGUU |
| mir-195-5p mimics | Sense | UAGCAGCACAGAAAUAUUGGC |
|  | Antisense | CAAUAUUUCUGUGCUGCUAUU |
| mir-497-5p mimics | Sense | CAGCAGCACACUGUGGUUUGU |
|  | Antisense | AAACCACAGUGUGCUGCUGUU |
| Negative control | Sense | UUCUCCGAACGUGUCACGUTT |
|  | Antisense | ACGUGACACGUUCGGAGAATT |
| mir-424-5p inhibitor |  | UUCAAAACAUGAAUUGCUGCUG |
| Inhibitor NC |  | CAGUACUUUUGUGUAGUACAA |
| GAPDH | Forward primer | GCACCGTCAAGGCTGAGAAC |
|  | Reverse primer | TGGTGAAGACGCCAGTGGA |
| TRPM2-AS | Forward primer | CACTTACTCATCCAAGAACCCACC |
|  | Reverse primer | GCCAACCCACAAGGAGACAGG |
| PDIA6 | Forward primer | GGGGATTGGAGGGTTTGGGTA |
|  | Reverse primer | CTCCCTGAGAAACTCGTTGATGC |
| mir-424-5p | Forward primer | GCCAGCAGCAATTCATGT |
|  | Reverse primer | TATGGTTTTGACGACTGTGTGAT |
| U6 | Forward primer | CAGCACATATACTAAAATTGGAACG |
|  | Reverse primer | ACGAATTTGCGTGTCATCC |
