# Supplementary figures and images for: PDIA6, which is regulated by TRPM2-AS/miR-424-5p axis, promotes endometrial cancer progression via TGF-beta pathway
Source: Cell Death Dis. 2023 Dec 14;14(12):829. doi: 10.1038/s41419-023-06297-8 (PMC10721792; doi:10.1038/s41419-023-06297-8)

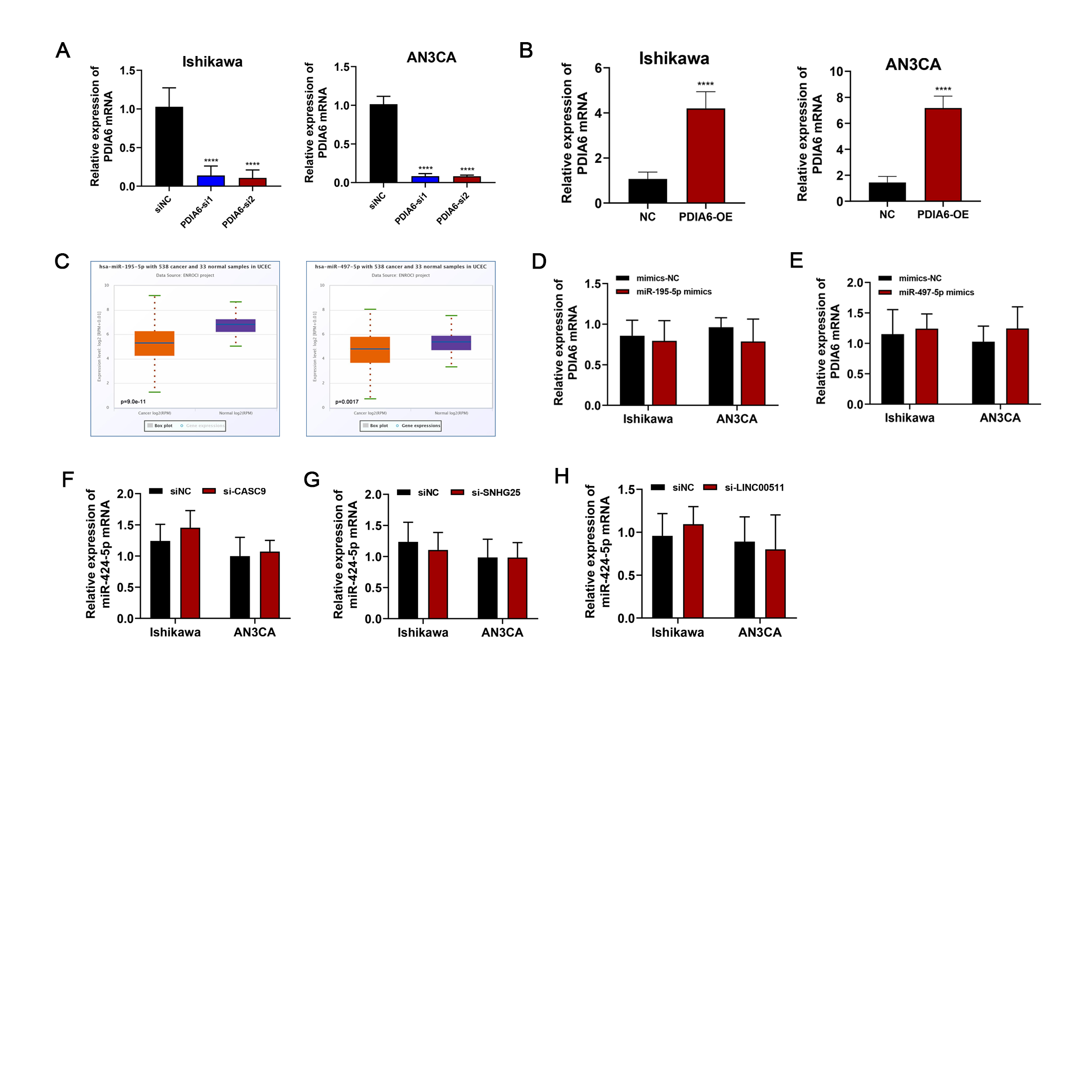

Supplement: Supplementary file 3 — Supplymentary Figure 1 [file 41419_2023_6297_MOESM3_ESM.tif]

Pengling Wang


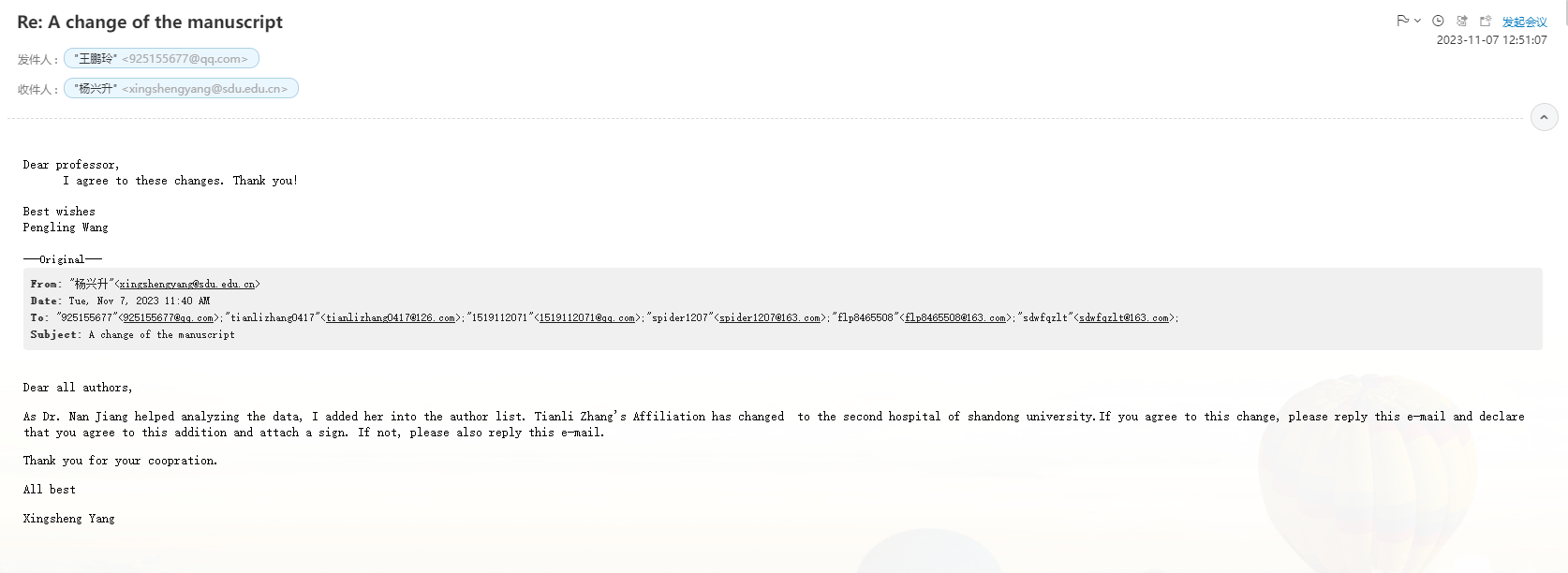


Tianli Zhang


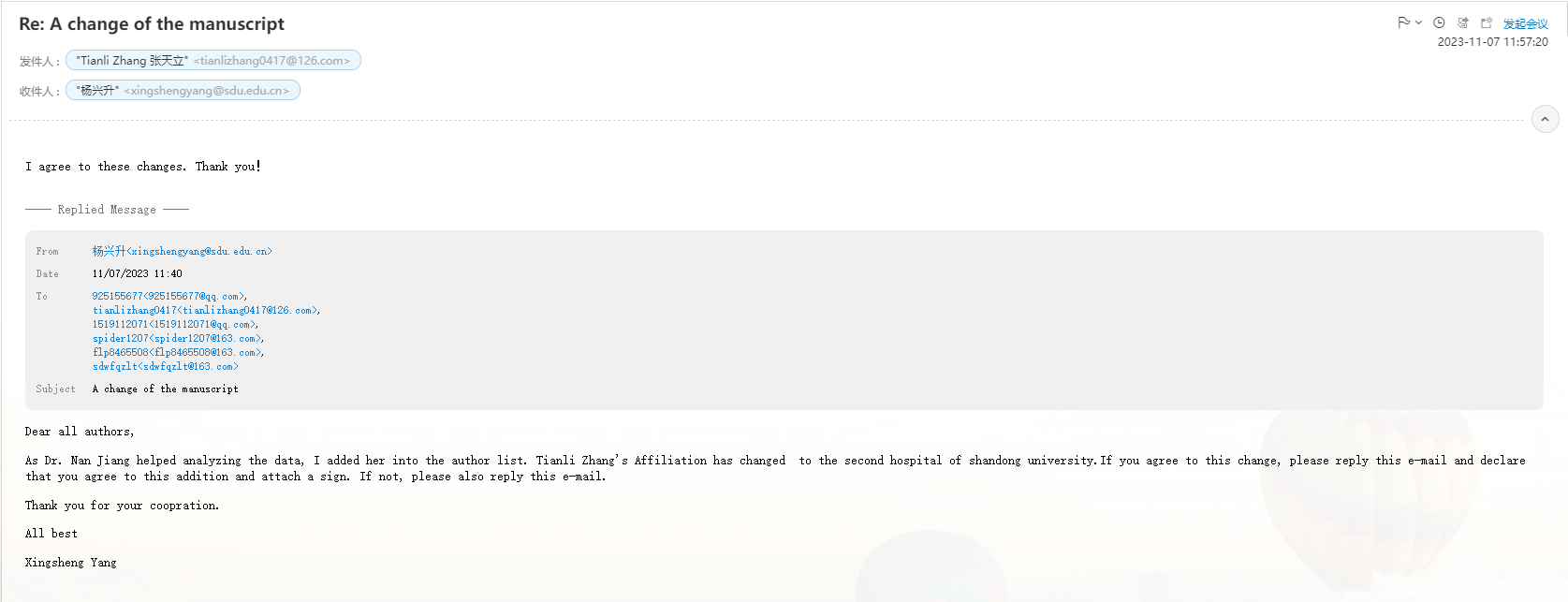


Nan Jiang


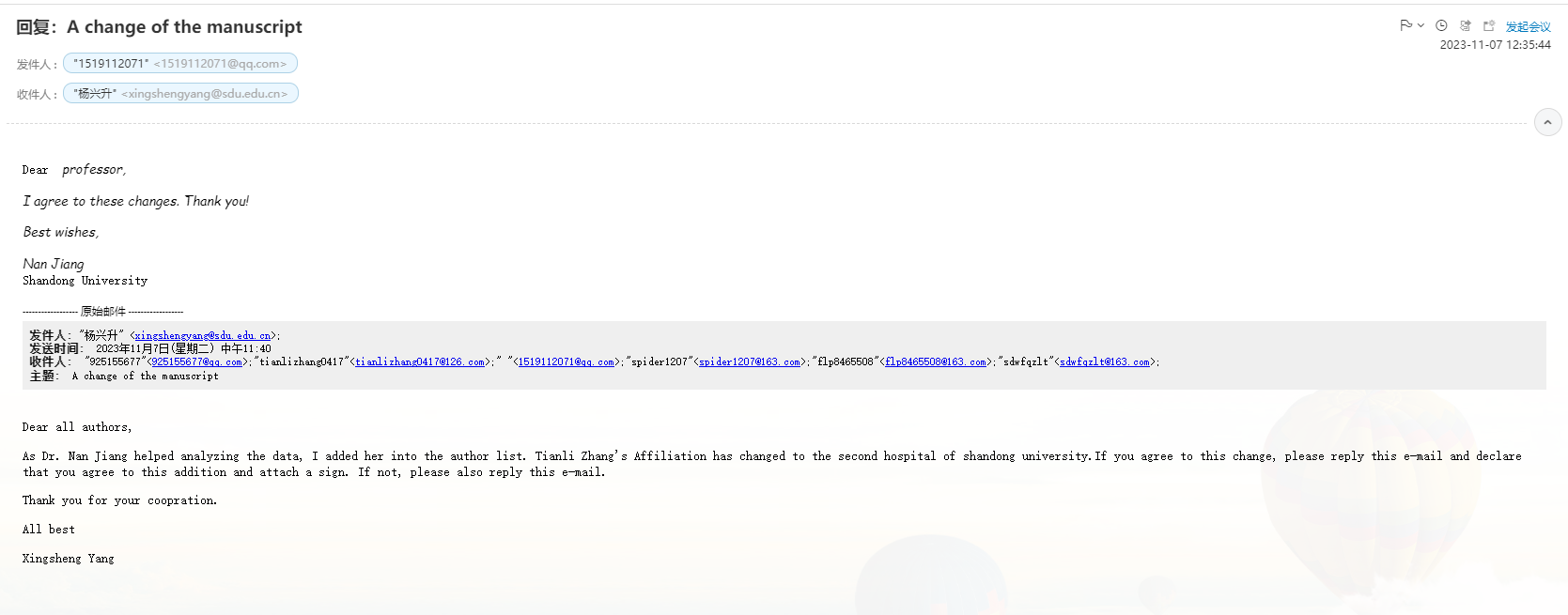


Kun Wang


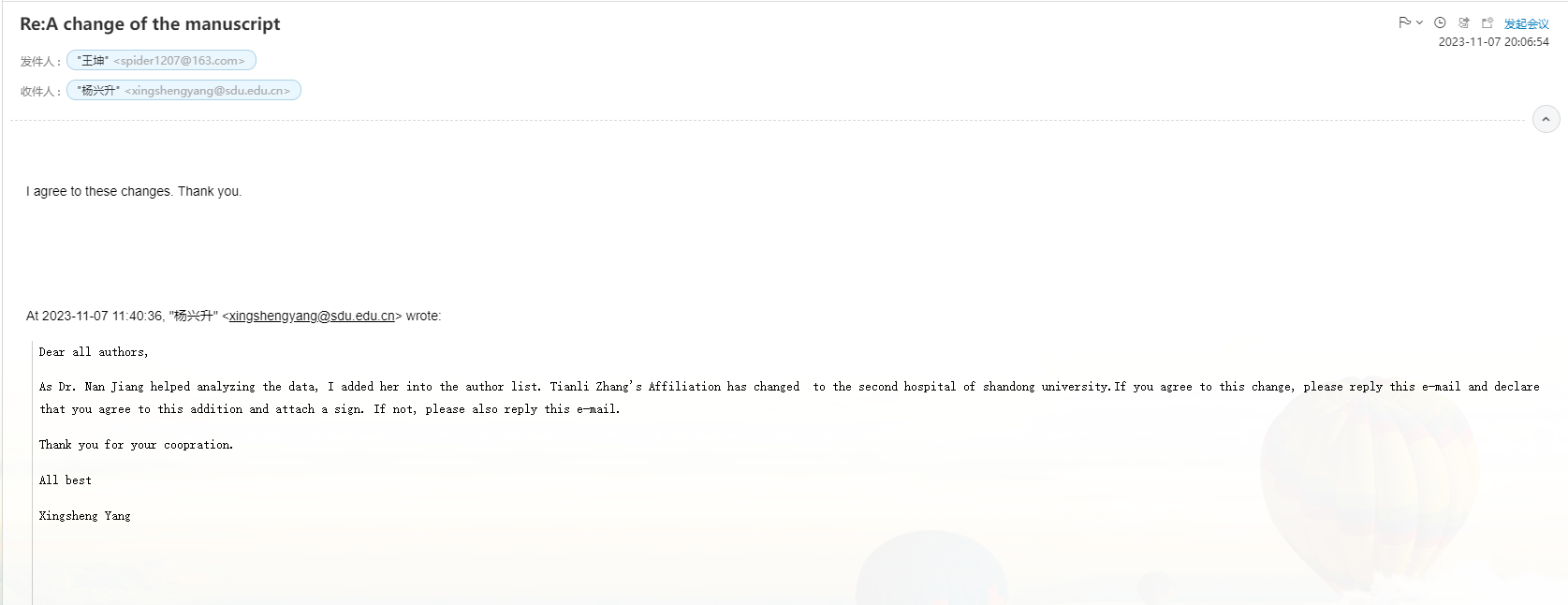


Liping Feng


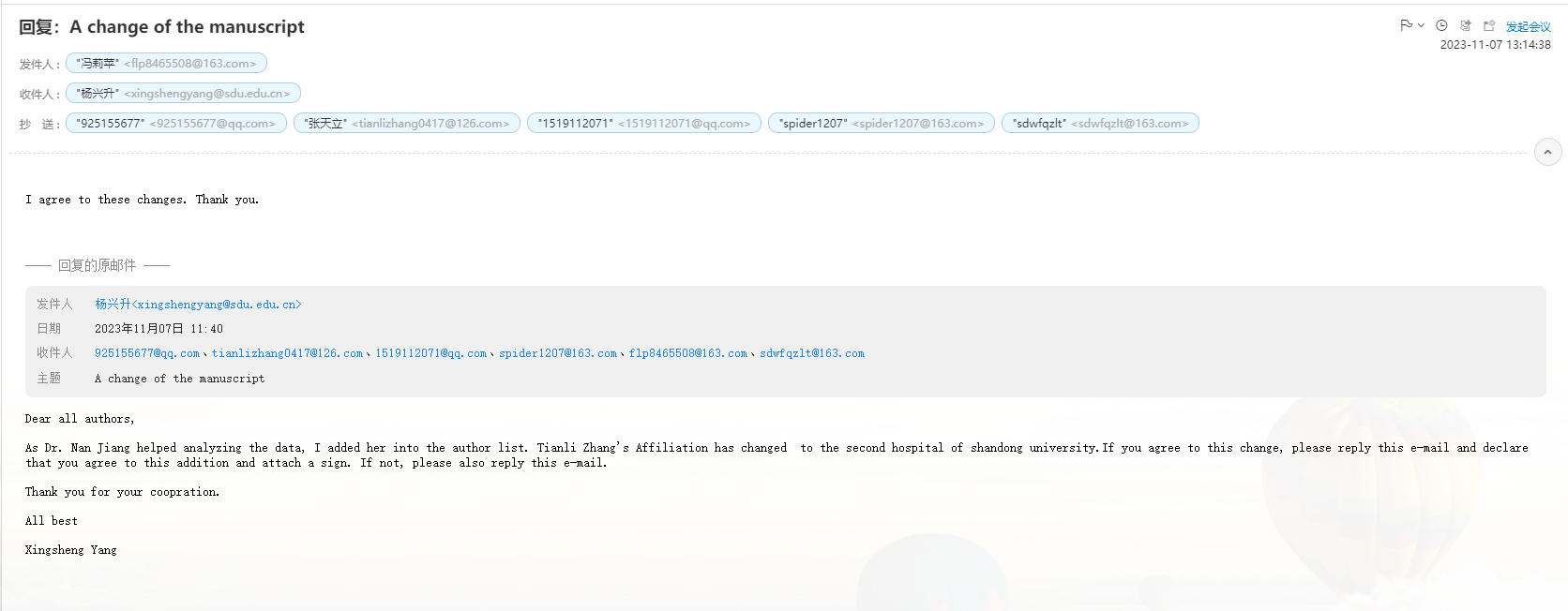


Ting Liu


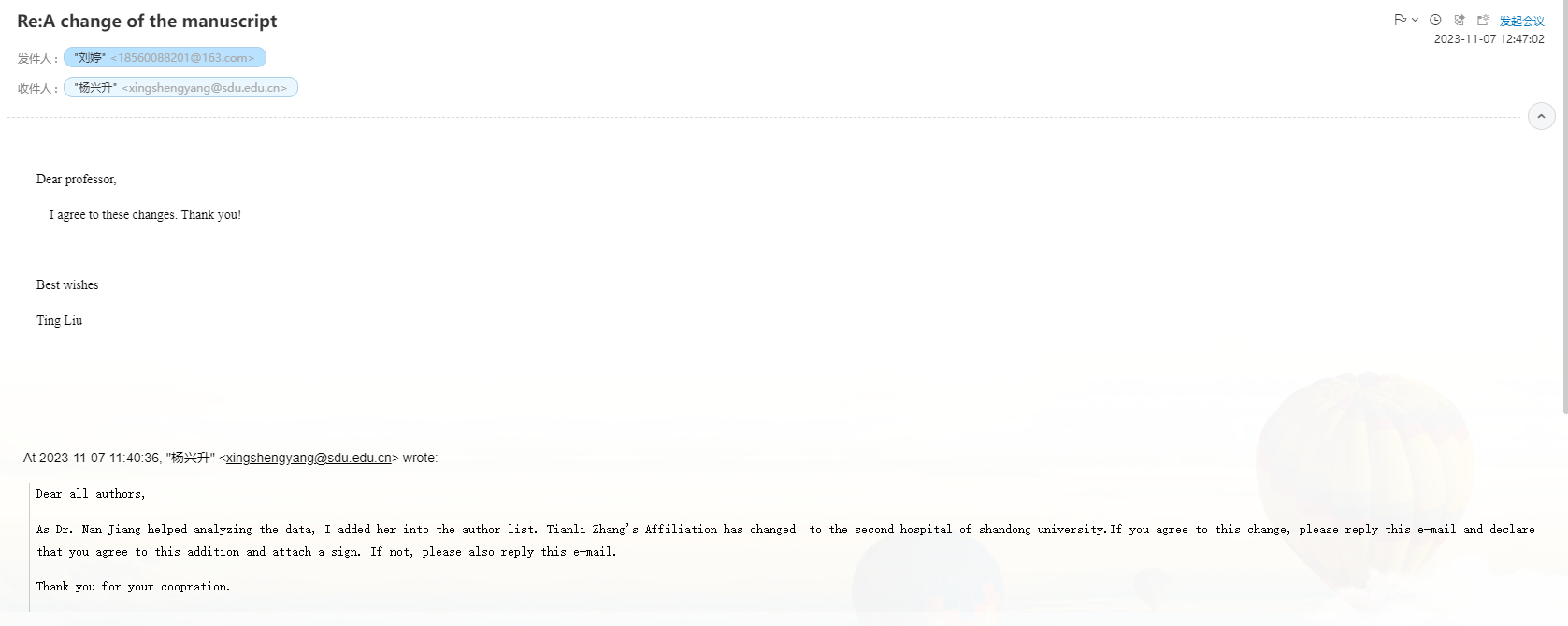

Supplement: Supplementary file 5 — A change of the manuscript [file 41419_2023_6297_MOESM5_ESM.docx]
