# Supplementary material for: PDIA6, which is regulated by TRPM2-AS/miR-424-5p axis, promotes endometrial cancer progression via TGF-beta pathway
Source: Cell Death Dis. 2023 Dec 14;14(12):829. doi: 10.1038/s41419-023-06297-8 (PMC10721792; doi:10.1038/s41419-023-06297-8)
Supplement: Supplementary file 4 — Original Data File [file 41419_2023_6297_MOESM4_ESM.pdf]

Original data of western blot

A

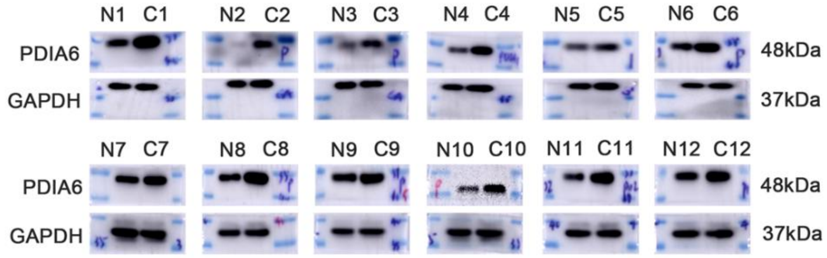

B

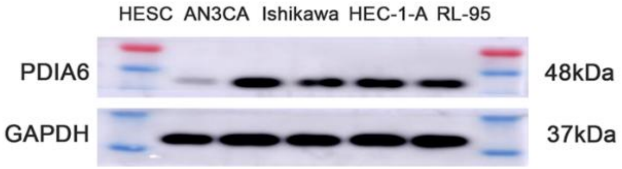

C

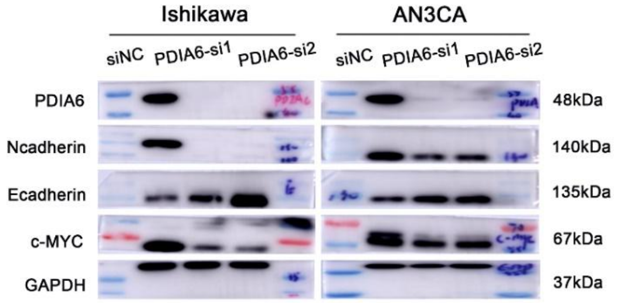

D

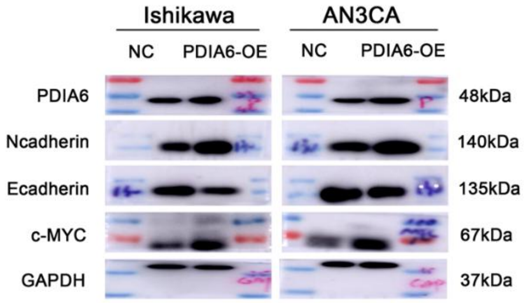

E

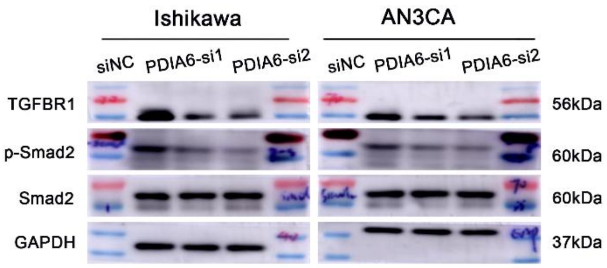

F

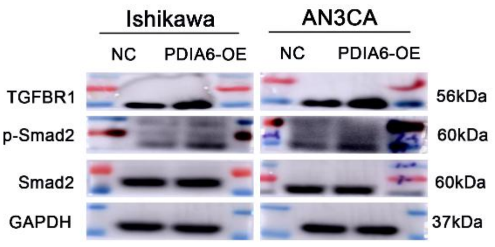

G

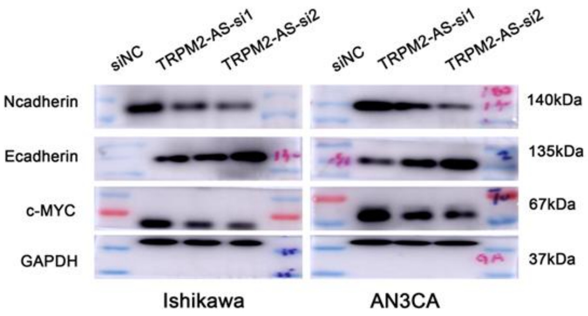

H

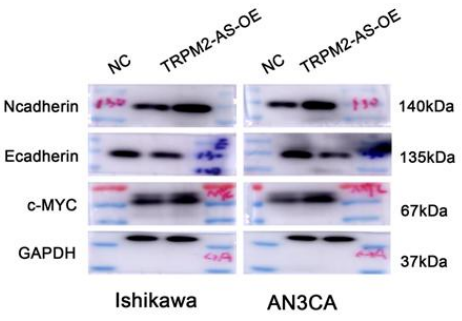

I

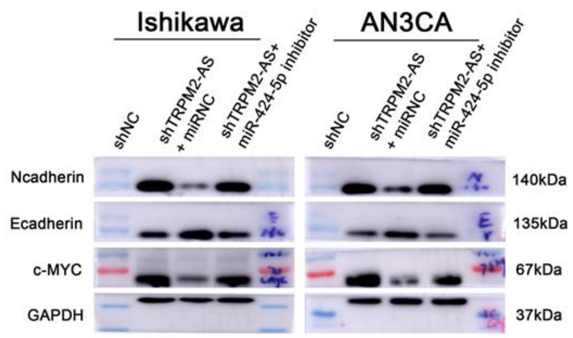

J

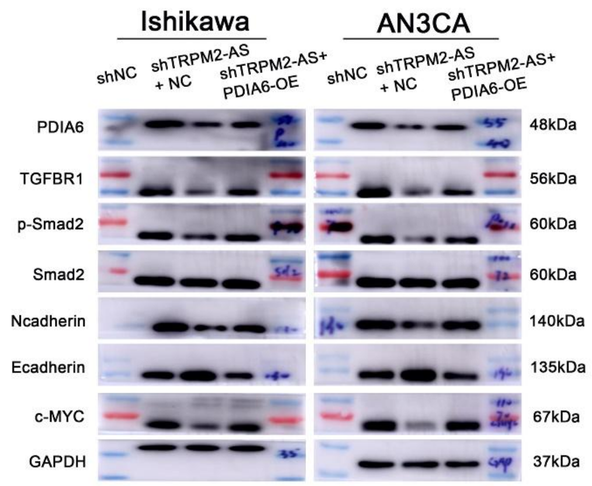

A. Fig.1（F）;
B. Fig.1（H）;
C. Fig.3（C）;
D. Fig.3（D）;
E. Fig.3（E）;
F. Fig.3（F）;
G. Fig.5（J）;
H. Fig.5（K）;
I. Fig.6（D）;
J. Fig.7（F）。
